# Prepulse Inhibition in Cocaine Addiction and Dual Pathologies

**DOI:** 10.3390/brainsci11020269

**Published:** 2021-02-20

**Authors:** Isis Gil-Miravet, Alejandro Fuertes-Saiz, Ana Benito, Isabel Almodóvar, Enrique Ochoa, Gonzalo Haro

**Affiliations:** 1 TXP Research Group, Universidad Cardenal Herrera-CEU, CEU Universities, 12006 Castellón, Spain; isis.gil@uji.es (I.G.-M.); anabenitodel@hotmail.com (A.B.); isabel.almodovar@uchceu.es (I.A.); gonzalo.haro@uchceu.es (G.H.); 2 Predepartamental Unit of Medicine, Universitat Jaume I, 12071 Castellón, Spain; 3 Psychiatry Department, Consorcio Hospitalario Provincial de Castellón, 12002 Castellón, Spain; 4 Torrente Mental Health Centre, Hospital General Universitario, 46014 Valencia, Spain; 5 Molecular Biopathology Department, Consorcio Hospitalario Provincial de Castellón, 12002 Castellón, Spain; enrique.ochoa@hospitalprovincial.es

**Keywords:** dual diagnosis, schizophrenia, antisocial personality disorder, cocaine-related disorder, psychopathy, prepulse inhibition

## Abstract

Cocaine addiction is frequently associated with different psychiatric disorders, especially schizophrenia and antisocial personality disorder. A small number of studies have used prepulse inhibition (PPI) as a discriminating factor between these disorders. This work evaluated PPI and the phenotype of patients with cocaine-related disorder (CRD) who presented a dual diagnosis of schizophrenia or antisocial personality disorder. A total of 74 men aged 18–60 years were recruited for this research. The sample was divided into four groups: CRD (*n* = 14), CRD and schizophrenia (*n* = 21), CRD and antisocial personality disorder (*n* = 16), and a control group (*n* = 23). We evaluated the PPI and other possible vulnerability factors in these patients by using different assessment scales. PPI was higher in the CRD group at 30 ms (F(3, 64) = 2.972, *p* = 0.038). Three discriminant functions were obtained which allowed us to use the overall Hare Psychopathy Checklist Revised score, reward sensitivity, and PPI at 30 ms to predict inclusion of these patients in the different groups with a success rate of 79.7% (42.9% for CRD, 76.2% for CRD and schizophrenia, 100% for CRD and antisocial personality disorder, and 91.3% in the control group). Despite the differences we observed in PPI, this factor is of little use for discriminating between the different diagnostic groups and it acts more as a non-specific endophenotype in certain mental disorders, such as in patients with a dual diagnosis.

## 1. Introduction

Behind cannabis, cocaine is the second most widely used illicit drug in the EU. Around four million Europeans aged 15–64 used cocaine last year [1]. Cocaine addiction is frequently associated with several psychiatric disorders, especially schizophrenia (SCZ) and antisocial personality disorder (APD) [2,3], and other public health problems—often with serious social and economic consequences [4]. Moreover, cocaine users tend not to respond to treatments and often relapse [5,6,7]. However, not all cocaine users become addicted and not all patients with cocaine-related disorder (CRD) develop a psychiatric comorbidity, or dual pathology (DP) [3]. Identifying predisposing factors or endophenotypes for CRD and DPs would aid our understanding of vulnerabilities to CRD and DPs and help the development of risk factor-based prevention and treatment strategies [7,8,9,10,11].

Prepulse inhibition (PPI) of the acoustic startle reflex is widely used to measure sensorimotor activation and provide information on the processing capacity and function of patients [12,13]. PPI measures the reflex amplitude difference elicited by a “pulsed” sound stimulus subsequent to a less intense “prepulse”. PPI can be modulated by the interval (normally 30–300 ms) separating the two pulses, although maximum inhibition usually occurs at 100–120 ms [14,15]. Regarding intervals, shorter ones such as 30 ms respond to pre-attentive and automatic perceptual processing, whereas longer intervals such as 120 ms are partially automatic, meaning that they can proceed automatically, but also of controlled attentional modulation [16]. PPI is a good neurobiological marker between different species because it is hereditary and does not become habituated or eliminated over repeated tests [9,17,18,19,20]. Moreover, PPI is reduced in patients with schizophrenia, making it a good endophenotype for this pathology [21,22,23,24,25,26], and lower PPI is also associated with antisocial characteristics [27,28]; a recent study used PPI to discriminate between patients with APD alone or APD with psychopathy [29]. We also previously showed that patients with CRD + SCZ or CRD + APD presented PPI deficits compared to patients with CRD without a DP [30].

The cortico–striatal–pallido–thalamic circuitry of the forebrain and its pontine projections are thought to be the main regulators of PPI [31,32,33]. Interestingly, alterations in the nucleus accumbens (NAc), hippocampus, corpus striatum, globus pallidus, and thalamus—all related to the startle circuit—have also been noted in patients with schizophrenia [34]. Similarly, PPI appears to be modulated by central dopamine (DA)-dependent mechanisms. Amphetamines (indirect DA agonists) decreased PPI and increased DA in the NAc [35], while cocaine generated dopaminergic system neuroplasticity in the NAc [36]. In mice, baseline PPI also correlated with sensitivity to the reinforcing effects of cocaine in the conditioned place preference paradigm; animals with low PPI had higher striatal DA D2 receptor expression and required higher doses of cocaine (12 mg/kg) to acquire a conditioned preference than those with higher PPI [9]. Thus, DA availability in the NAc appears to be critical in determining the PPI; higher DA levels induce a PPI deficit, while lower DA levels increase PPI [21,35]. Together, these data suggest that patients with lower PPI levels present dopaminergic system alterations that increase their vulnerability to CRD.

Very few studies have used PPI to study substance use and its DPs in humans, despite their apparent shared biological bases. Thus, our objectives were to determine the relationship between PPI and CRD, either alone or as a DP with schizophrenia or APD, compared with healthy individuals and to examine if PPI can discriminate between controls and patients with CRD with or without schizophrenia or APD.

## 2. Materials and Methods

A total of 74 patients were recruited from the Addictive Behavior Unit, Hospital Detoxification Unit, or Severe Dual Diagnosis Program at the Provincial Consortium Hospital, Castellón (Spain), while receiving treatment for a CRD. The inclusion criteria for the experimental group were: (a) men aged 18–60 years; (b) a diagnosis of CRD alone or as a DP with schizophrenia or APD; (c) cocaine use in the last 30 days; (d) the absence of other mental disorders. Control participants were recruited through hospital open days and the inclusion criteria were: (a) men aged 18–60 years; (b) absence of substance use disorders; (c) absence of mental disorders; (d) absence of mental disorders in first-degree relatives. The participants were divided into four groups: (1) controls (*n* = 23; age = 42.39 ± 10.67); (2) CRD (*n* = 14; age = 44.64 ± 5.54); (3) CRD + SCZ and (*n* = 21; age = 38.57 ± 7.78); and (4) CRD + APD (*n* = 16; age = 42.88 ± 5.88). The patient sociodemographic characteristics are shown in Table 1.

This study protocol was approved by the Ethics Committee at the Castellón Provincial Hospital Consortium, considering the ethical principles established in the Declaration of Helsinki. The confidentiality of the participants and the data was always guaranteed. All the participants were informed about the study verbally and in writing and then signed their written informed consent before the work started. A sociodemographic questionnaire created specifically for this study was then administered.

The experimental patients completed the Dual Diagnostic Screening Interview [37] to detect the most frequent comorbid psychiatric diagnoses in substance abusers, and the Psychiatric Research Interview for Substance and Mental Disorders (PRISM-IV) [38], which assesses psychiatric pathologies and past and current disorders caused by substance use. Control participants completed the MINI International Neuropsychiatric Interview (MINI 5.0.0) [39] to diagnose the main axis I of the Diagnostic and Statistical Manual of Mental Disorders, Fourth Edition (DSM-IV) and the International Classification of Diseases, Tenth Revision (ICD-10) psychiatric disorders. All the participants completed the Levenson Self-Report Psychopathy Scale (LSRP) [40] and the revised Hare Psychopathy Checklist Revised (PCL-R) [41] to assess psychopathy and interpersonal/affective and social deviance, with psychopathy diagnosed with total PCL-R scores exceeding 26 [29]. Finally, the Sensitivity to Punishment and Sensitivity to Reward Questionnaire (SPSRQ) [42] and Barrat Impulsiveness Scale (BIS) [43] were used to measure impulsivity.

We measured PPI following a previously published protocol [30] using a BIOPAC MP 150 QUICK START (Mark II, SR-Lab, San Diego, California) system to generate a weak prepulse (a consciously imperceptible sound) preceding an intense “pulse” startle sensory stimulus and measuring the startle response with orbicular electromyography (measured in millivolts). Patients were acclimatized to white noise at 70 dB and then received three blocks of stimuli: the first and last blocks were identical and comprised five pulses at 105 dB for 40 ms; the second block comprised eight pulses and 24 pulses with a prepulse at 30, 60, or 120 ms at 85 dB for 20 ms. A total of 42 trials were performed over approximately 15 min. The main dependent variable was the percentage of PPI calculated as: {[(single pulse response) − (pulse with prepulse response)] ÷ (single pulse response)} × 100. The mean latency, amplitude, and habituation were also calculated. The researchers performing these tests were trained to administer and evaluate them, and they were administered when psychopathological stability was achieved after 10 days of patient detoxification. Furthermore, patients could not smoke or drink coffee for at least one hour before PPI was measured.

All the statistical analyses were performed using SPSS software (v. 21, IBM Corp., Armonk, NY). Exploratory analysis of the data was carried out by performing tests of normality (Q–Q graphics, Kolmogorov–Smirnov, and Shapiro–Wilk) and homoscedasticity (Levene test). The main sociodemographic variables were compared between the groups using Chi-squared tests and are shown as the mean ± SD. The PPI intervals between groups were first compared using mixed ANOVA, followed by one-way ANOVAs with Tukey or Games–Howell post-hoc tests (according to the homogeneity of the variances) to analyze differences in the daily antipsychotics doses, sensitivity to punishment and reward, psychopathy, and PPI variables. Pearson correlations were performed to compare the daily antipsychotics doses and PPIs. Finally, discriminant analyses were implemented to check whether these variables could differentiate the four sub-groups.

## 3. Results

The mean participant age was 41.84 ± 8.27 years and did not significantly differ between the groups (*F*(3, 69) = 1.805, *p* = 0.154). As shown in Table 1, we found significant differences for marital status (χ2 = 37.672, *p* < 0.001), living arrangement (χ2 = 42.298, *p* = 0.001), educational level (χ2 = 48.038, *p* < 0.001), and employment status (χ2 = 81.262, *p* < 0.001), but not in the number of children (χ2 = 16.578, *p* = 0.056). Patients with CRD were more frequently separated (adjusted standardized residuals (ASR) = 3.0) and the CRD + SCZ group members were more often single (ASR = 2.9) than married (ASR = −2.5) compared to the controls (ASR = −2.9 and 4.9, respectively). The CRD + SCZ group more often lived with friends (ASR = 2.2) or their parents (ASR = 2.7) compared to controls (ASR = −2.8). The CRD + APD group more frequently had other living arrangements (ASR = 2.2). Both the CRD + SCZ and CRD + APD groups tended not to live with a partner and children (ASR = −2.6 and −2.0, respectively, vs. control, ASR = 4.7).

The CRD group had often not completed secondary school (ASR = 2.7), while both the CRD + SCZ and CRD + APD groups more often had not completed primary school (ASR = 2.5 for both) compared to the controls (ASR = −3.3), who had more often completed a bachelor’s (ASR = 2.4) or advanced degree/doctorate (ASR = 4.5). CRD patients more frequently had unpaid family business employment (ASR = 2.1) or temporary employment (ASR = 2.0), and the CRD + SCZ group were more often pensioners or had a permanent disability (ASR = 4.7) compared to controls (ASR = −3.9). The CRD + APD group were more frequently unemployed but had previously worked (ASR = 2.7 vs. controls ASR = −3.3). Compared to controls (ASR = 6.1), who were often students or studying for public servant exams (ASR = 2.6), both the CRD + SCZ and CRD + APD groups had fewer self-employed or permanently employed patients (ASR = −2.1 and −3.3, respectively).

There was a mean of 3.29 ± 1.61 addictions across the addict groups, with significant differences (F(2, 48) = 6.376, *p* = 0.004); the CRD + APD group had the most addictions (*p* = 0.009 and 0.008, respectively) and the addiction severity did not differ between the addict groups (F = 0.325, *p* = 0.724). However, the ages at cocaine use onset (F = 3.971, *p* = 0.025) and CRD onset (F = 6.649, *p* = 0.003) did vary, with consumption having started later in the CRD group than the CRD + SCZ group (*p* = 0.034) and the age at addiction onset being higher in the CRD group than for the CRD + SCZ (*p* = 0.004) and CRD + APD (*p* = 0.009) groups. Table 2 shows these data and the percentage of subjects addicted to each substance. Supplementary Table S1 shows more characteristics of addictions (age at onset, route of administration, and weekly frequency of use).

In total, 51.1% (*n* = 24) of patients used antipsychotics. The mean daily antipsychotic drug dose (converted to chlorpromazine) was 70.04 ± 59.71 mg/day. Differences between groups in total antipsychotics and quetiapine doses can be seen in Table 2. Data about other antipsychotics can be seen in Supplementary Table S2.

PPI did not correlate with the daily dose of antipsychotics in any group, but in the CRD + SCZ group, there was a strong negative correlation between PPI at 30 ms and the daily dose of quetiapine (*n* = 14, *r* = −0.599, *p* = 0.04). PPI did not correlate with the antipsychotic dose in the other groups. In the CRD + SCZ group, no differences were observed between PPI at 30 ms (mean = −0.37 ± 23.26) compared to the control group (0.8 ± 32.47, *t* = −0.930, *p* = 0.926) for patients whose daily quetiapine dose did not exceed the recommended 600 mg (*n* = 8), while the mean PPI was lower (−33.48 ± 34.63 vs. the control at 0.8 ± 34.63, *t* = −2.274, *p* = 0.031) in patients on higher quetiapine doses (*n* = 6).

As shown in Table 3, the ANOVA analysis showed significant differences both for the primary and secondary LSRP scores, and Tukey tests showed lower control group scores compared to the CRD + SCZ (*p* < 0.001), CRD + APD (*p* = 0.004), and CRD (*p* < 0.001) groups. The secondary scores were significantly lower in the CRD and control groups compared with the CRD + SCZ (*p* = 0.031 and *p* < 0.001) and CRD + APD (*p* = 0.038 and *p* < 0.001) groups. Again, ANOVA analyses showed significant differences in PCL-R scores for all the subscales, which were lower for the control group social deviation factor (*p* = 0.004 for CRD and *p* < 0.001 for CRD + SCZ and CRD + APD) and for CRD and CRD + SCZ compared to CRD + APD (*p* < 0.001). Similarly, the control scores were significantly lower than those of the CRD + SCZ, CRD + APD, and CRD groups (*p* < 0.001) for the interpersonal/affective factor; CRD and CRD + SCZ were lower than CRD + APD (*p* < 0.001), and CRD was lower than CRD + SCZ (*p* = 0.027). The overall score was significantly lower in the controls compared to the other groups (*p* < 0.001) and in the CRD and CRD + SCZ groups compared to the CRD + APD group (*p* < 0.001). Together, these results suggest the following ascending psychopathy pattern: control < CRD + SCZ ≈ CRD < CRD + APD, although CRD + SCZ scores were significantly higher than CRD in the secondary LSRP factor and the PCL-R interpersonal/affective factor. One-way ANOVA also revealed significant differences in the SPSRQ results, with post-hoc tests showing higher scores for punishment sensitivity in the CRD + SCZ group compared to the CRD (*p* = 0.005), CRD + APD (*p* = 0.036), and control (*p* = 0.001) groups.

**Figure 1:**
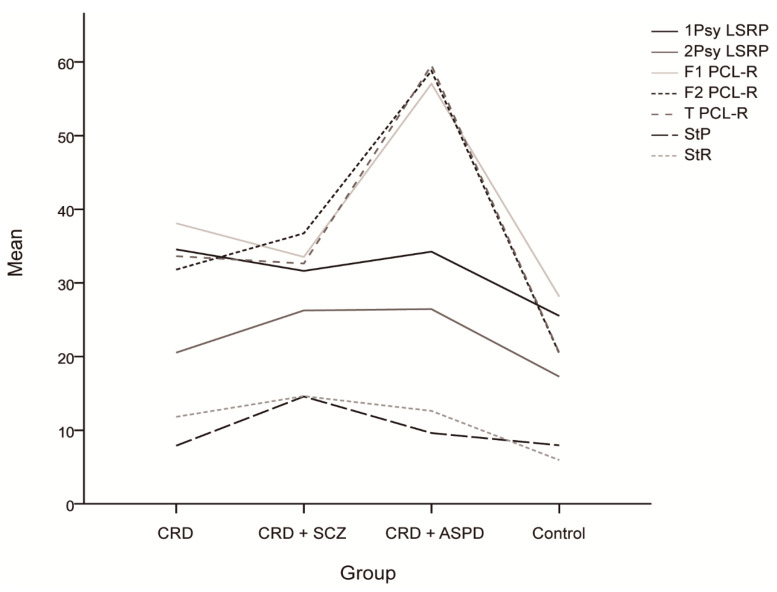
Mean scores and differences between the groups for the psychopathy scales and sensitivity to punishment and sensitivity to reward questionnaire. CRD: cocaine-related disorder; SCZ: schizophrenia; APD: antisocial personality disorder; 1Psy LSRP: primary psychopathy on the Levenson Self-Report Psychopathy Scale; secondary psychopathy on the Levenson Self-Report Psychopathy Scale; F1 PCL-R: interpersonal/affective factor on the Hare Psychopathy Checklist Revised; F2 PCL-R: social deviation factor on the Hare Psychopathy Checklist Revised; T PCL-R: total score on the Hare Psychopathy Checklist Revised; StP: sensitivity to punishment; StR: sensitivity to reward.

**Figure 2:**
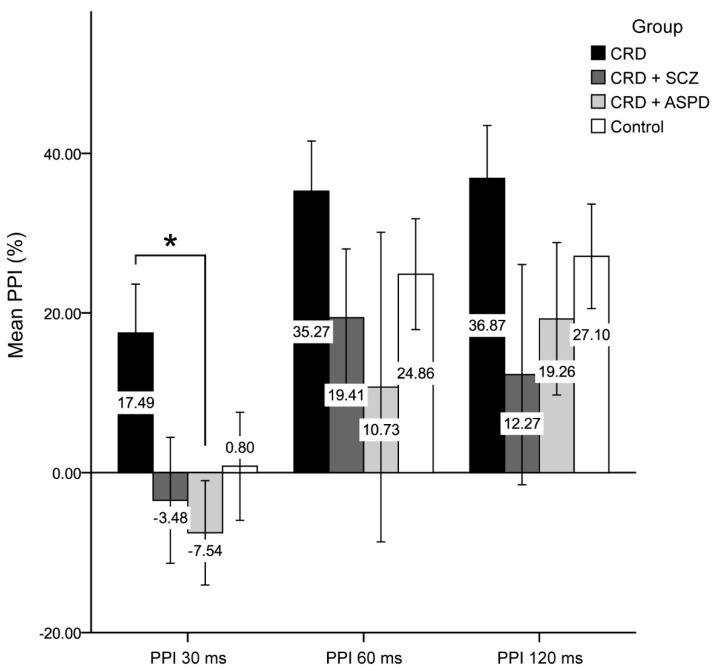
Prepulse inhibition percentage. CRD: cocaine-related disorder; SCZ: schizophrenia; APD: antisocial personality disorder; PPI: prepulse inhibition; ms: milliseconds. Data are shown as the mean ± standard deviation (* *p* < 0.05).

The differences in psychopathy between the groups are shown in Figure 1. The scores for the reward sensitivity factor were significantly lower in the control group compared to the CRD + SCZ (*p* < 0.001), CRD + APD (*p* < 0.001), and CRD (*p* = 0.007) groups. In the mixed ANOVA, neither a main group effect nor a group*PPI interval interaction effect was observed, although the PPI at 30 ms was lower than at 60 or 120 ms (F(2) = 11.816, *p* < 0.001). Figure 2 shows the PPI at 30, 60, and 120 ms, which was only significantly different for PPI at 30 ms using a one-way ANOVA (F(3, 64) = 2.972, *p* = 0.038). The post-hoc tests also found differences between the CRD and CRD + APD groups for PPI at 30 ms (*p* = 0.041) and PPI amplitude (F(3, 70) = 3.985, *p* = 0.011), but not for mean PPI (F(3, 62) = 1.273, *p* = 0.291) or habituation (F(3, 70) = 1.851, *p* = 0.146).

We obtained three discriminant functions that predicted patient inclusion in the groups with a 79.7% success rate using the scores for overall PCL-R, reward sensitivity, and PPI at 30 ms (42.9% CRD, 76.2% CRD + SCZ, 100% CRD + APD, and 91.3% controls). The first had an eigenvalue of 15.684 and explained 96.3% of the variance, the second was 0.435 at 2.7%, and the third was 0.162 at 1%. These equations were:

Z1: 0.974 × Total PCL-R + 0.139 × Reward Sensitivity − 0.027 × PPI 30

Z2: −0.087 × Total PCL-R + 0.966 × Reward Sensitivity + 0.324 × PPI 30

Z3: 0.209 × Total PCL-R − 0.219 × Reward Sensitivity + 0.946 × PPI 30

**Figure 3:**
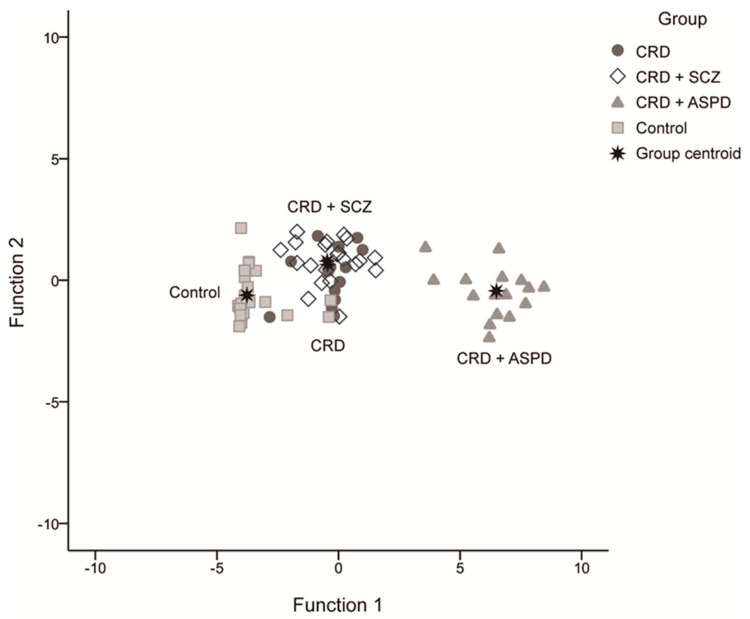
Scatter diagram for the discriminating functions. CRD: cocaine-related disorder; SCZ: schizophrenia; APD: antisocial personality disorder.

Figure 3 shows the scatter diagram for these discriminating functions.

## 4. Discussion

Our primary objective was to devise an explanatory model for the differences in PPI between patients with a DP CRD. The main effect of the PPI interval masked the differences between groups in the mixed ANOVA but could be identified using one-way ANOVA. These results indicated that individuals with a DP, especially CRD + APD, had deficient PPIs. Previous work had already shown that PPI could be a useful endophenotype for schizophrenia [18], but research regarding PPI and APD [28,30] or CRD DPs with these two pathologies is currently scarce or absent. Although we found no significant differences between the control and DP groups, PPI in this work was lower in patients with a DP, perhaps because PPI was likely modulated by the use of an atypical antipsychotic drug (quetiapine) in the CRD + SCZ group [44,45].

Quetiapine can reverse alterations in sensory filtering by regulating the dopaminergic system [46,47] and is negatively correlated with PPI at 30 ms. Thus, high doses of sedative antipsychotics counteract sensory filtering and could perhaps explain why, compared to the control group, the mean PPI at 30 ms remained stable in patients using doses up to the recommended amount of 750 mg [48] but was significantly lower in those using higher doses. In contrast, typical antipsychotics do not reverse these filtering alterations [49]. Hence, patients in the CRD group had much higher PPIs, although this was not significant compared to the control group.

Therefore, we hypothesize that, (1) in agreement with previous work, the neurocircuits associated with the startle reflex are altered in patients with APD [27,28] or schizophrenia [13,18,23,34,50,51], and therefore, cocaine would not increase their PPI [30]; (2) high doses of atypical sedative-type antipsychotics (e.g., quetiapine) can counteract the sensory filtering regulatory effects present in patients with schizophrenia; (3), cocaine consumption in patients without a DP—i.e., with a normal startle reflex—improves PPI when atypical antipsychotics are used.

Several murine studies have indicated that PPI is modulated by DA [52,53,54,55,56] and that schizophrenia is characterized by dopaminergic dysfunction in the striatum [57,58]. This is consistent with the PPI deficits we observed in DP groups, therefore suggesting altered dopaminergic functioning in these patients and that indirect DA agonists such as amphetamines and cocaine could decrease PPI. Indeed, amphetamines do decrease PPI, but cocaine—which increases DA in the NAc in similar proportions to amphetamines—does not; this may be because these substances have different mechanisms of action, as supported by the fact that amphetamines induce psychosis more frequently than cocaine [35]. Furthermore, cocaine only affected PPI in rats that were susceptible to apomorphine—another indirect DA agonist [59]. Moreover, mice acquired cocaine conditioning linearly with increasing PPI [9], and animals with low PPI were less sensitive to the conditioned rewarding effects of cocaine [60]. This could be related to DA D1 and D2 receptors in the striatum, because D2 receptor expression was higher in mice with low PPI [9].

These results might be the biological basis of the differences in PPI we found between patients with CRD + APD versus CRD alone. Assuming that the dopaminergic pathways of individuals with APD or schizophrenia are altered, resulting in lower PPI and higher D2 receptor expression, the intrinsic mechanisms of action of cocaine are then unlikely to themselves alter PPI. Cocaine blocks DA reuptake pumps, increasing the amount of DA present and inducing molecular changes such as cyclic adenosine monophosphate (cAMP) activation via D1 receptor activation [61,62]. The cAMP pathway—itself implicated in the expression of early-acting genes in the NAc—is involved in the development of compulsive behavior and formation of the characteristic habits of CRD [61,62]. Thus, variance in D1 and D2 receptor expression could account for the differences in PPI-related pathways in CRD patients with or without DPs, helping to explain why cocaine did not affect PPI in individuals with a DP.

Our secondary objective was to determine if PPI could discriminate between the different patient groups. As expected, the sociodemographic variables significantly differed between these groups, probably in relation to the specificities of these pathologies and CRD. Hence, the controls were usually married, lived with a partner and children, had high education levels, and were employed, while most CRD patients were single or divorced, lived alone, had an incomplete secondary education, and were unemployed. Patients with a DP were usually single, lived with their parents, had an incomplete basic education, and were unemployed or had a disability. Similar findings have been reported elsewhere and seemed to be influenced by drug consumption patterns, with patients with a DP usually having started using cocaine and becoming addicted earlier and presenting a wider range of polydrug use profiles [63,64,65]. Furthermore, in line with our results, scales for measuring psychopathy and reward and punishment sensitivity phenotypes were the best tools for making a differential diagnosis. Nonetheless, the observed differences in PPI did not discriminate well between the different diagnostic groups.

In summary, PPI can act as a non-specific endophenotype in APD and schizophrenia. Both epigenetic factors and specific endophenotypes act upon the dopaminergic system to encourage PPI deficits to evolve in different disorders (phenotypes), which are best differentiated by their specific clinical characteristics [19]. Cocaine can increase PPI in patients without a DP but fails to do so in cases with significant deficits in that endophenotype. Moreover, certain antipsychotics reverse the sensory filtering and dopaminergic system dysregulation present in schizophrenia [46,58,66]. Finally, like other studies examining PPI, this work had some limitations. First, to avoid possible biases, we only included men because PPI varies according to sex [27,67]. Second, high polyconsumption may have hindered the assignment of PPI results to cocaine use, although polyconsumption is representative of the general population, thus facilitating the extrapolation of our results [68]. Lastly, neither the degree of craving nor the cognitive state of the subjects were assessed, and this could have influenced the PPI data.

## 5. Conclusions

PPI deficiency is proposed as a DP endophenotype in patients with CRD and antisocial personality disorder. Furthermore, in the absence of comorbidities and, therefore, in the absence of these endophenotypes, cocaine use increases PPI. Although PPI as an endophenotype has little utility in the differential diagnosis of CRD DPs, it may represent a non-specific target for future treatments, especially those which exert their action upon the dopaminergic system.

## Figures and Tables

**Table 1:** Sociodemographic descriptions.

Variables	Total	Control	CRD	CRD+SCZ	CRD+APD	χ^2^ (Sig.)
**Marital status**						
Single	44 (59.5%)	8 (34.5%)	6 (42.9%)	18 (85.7%)	12 (75%)	37.672 (0.000) *
Married	18 (24.3%)	14 (60.9%)	2 (14.3%)	1 (4.8%)	1 (6.3%)	
Separated	2 (2.7%)	0	2 (14.3%)	0	0	
Divorced	10 (13.5%)	1 (4.3%)	4 (28.6%)	2 (9.5%)	3 (18.8%)	
**Current living arrangement**						
Alone	19 (25.7%)	5 (21.7%)	5 (35.7%)	5 (23.8%)	4 (25%)	42.290 (0.001) *
Only with children	1 (1.4%)	0	0	0	1 (6.3%)	
With parents	19 (25.7%)	1 (4.3%)	3 (21.4%)	10 (47.6%)	5 (31.3%)	
As a couple without children	5 (6.8%)	2 (8.7%)	2 (14.3%)	0	1 (6.3%)	
As a couple with children	19 (25.7%)	14 (60.9%)	3 (21.4%)	1 (4.8%)	1 (6.3%)	
With friends	6 (8.1%)	1 (4.3%)	0	4 (19%)	1 (6.3%)	
Other arrangement	5 (6.8%)	0	1 (7.1%)	1 (4.8%)	3 (18.8%)	
**Number of children**						
0	38 (55.1%)	9 (40.9%)	5 (35.7%)	17 (89.5%)	7 (50%)	16.578 (0.56)
1	14 (20.3%)	4 (18.2%)	5 (35.7%)	1 (5.3%)	4 (28.6%)	
2	14 (20.3%)	7 (31.8%)	3 (21.4%)	1 (5.3%)	3 (21.4%)	
3	3 (4.3%)	2 (9.1%)	1 (7.1%)	0	0	
**Education**						
Incomplete primary education	23 (31.1%)	1 (4.3%)	2 (14.3%)	11 (52.4%)	9 (56.3%)	48.038 (0.000) *
Incomplete vocational training	10 (13.5%)	3 (13%)	5 (35.7%)	1 (4.8%)	1 (6.3%)	
Non-compulsory secondary education	21 (28.4%)	4 (17.4%)	5 (35.7%)	6 (28.6%)	6 (37.5%)	
Incomplete university bachelor’s degree	7 (9.5%)	3 (13%)	1 (7.1%)	3 (14.3%)	0	
University bachelor’s degree	5 (6.8%)	4 (17.4%)	1 (7.1%)	0	0	
Advanced university graduate or doctorate degree	8 (10.8%)	8 (34.8%)	0	0	0	
**Employment status**						
Other arrangement	1 (1.4%)	0	0	1 (4.8%)	0	81.262 (0.000) *
Student or studying for public servant exams	3 (4.1%)	3 (13%)	0	0	0	
Permanent disability or pensioner	23 (31.1%)	0	2 (14.3%)	15 (71.4%)	6 (37.5%)	
Unemployed, having previously worked	18 (24.3%)	0	5 (35.7%)	5 (23.8%)	8 (50%)	
Unemployed, not having previously worked	2 (2.7%)	0	1 (7.1%)	0	1 (6.3%)	
Unpaid employment in a family business	1 (1.4%)	0	1 (7.1%)	0	0	
On a temporary contract or temporary employment relationship	6 (8.1%)	3 (13%)	3 (21.4%)	0	0	
In a permanent employment relationship or contract, or self-employed	20 (27%)	17 (73.9%)	2 (14.3%)	0	1 (6.3%)	

Note: CRD: cocaine-related disorder; SCZ, schizophrenia; APD, antisocial personality disorder. * *p* < 0.01.

**Table 2:** Addictions and antipsychotic treatment.

Variables	CRD	CRD+SCZ	CRD+APD	*F/*χ^2^ (Sig.)
**Cocaine**				
Addiction % (*n*)	100 (14)	100 (21)	100 (16)	-
Addiction severity	7.14 (1.83)	7.67 (2.72)	7.75 (1.77)	0.32 (0.724)
Age at onset of use	**23.71 (6.62)**	**18.81 (4.03)**	18.88 (6.13)	3.97 (0.025) *
Age at onset of addiction	**27.64 (7.92)**	**20.24 (4.13)**	**20.44 (7.33)**	6.64 (0.003) **
Weekly use	4–6 days	Less than a day	Daily	13.98 (0.058)
Route of use	**Intranasal**	**Intranasal**	**Smoked/Injected**	11.08 (0.022) *
**Nicotine Addiction % (*n*)**	92.3 (12)	100 (20)	93.3 (14)	1.50 (0.470)
**Alcohol Addiction % (*n*)**	50 (7)	66.7 (14)	56.3 (9)	1.02 (0.671)
**Cannabis Addiction % (*n*)**	57.1 (8)	76.2 (16)	75 (12)	1.68 (0.462)
**Amphetamine Addiction % (*n*)**	7.1 (1)	9.5 (2)	25 (4)	2.54 (0.346)
**Heroin Addiction % (*n*)**	42.9 (6)	**19 (4)**	**87.5 (14)**	17.21 (<0.001) **
**Other opiates Addiction % (*n*)**	0	0	12.5 (2)	4.55 (0.166)
**Sedatives Addiction % (*n*)**	14.3 (2)	**14.3 (3)**	**62.5 (10)**	12.29 (0.002) **
**Antipsychotics**				
Treatment % (*n*)	25 (3)	**100 (19)**	**12.5 (2)**	50.48 (<0.001) **
Mean daily dose ^1^	0.45 (0.39)	90.09 (52.09)	0.11 (0.08)	6.88 (0.006) **
**Quetiapine**				
Treatment % (*n*)	33.3 (4)	**73.7 (14)**	**16.7 (2)**	10.77 (0.004) **
Mean daily dose	381.25 (251.14)	514.28 (292.48)	100 (70.71)	2.09 (0.154)

Note: CRD: cocaine-related disorder; SCZ: schizophrenia; APD: antisocial personality disorder. ^1^ Converted to chlorpromazine. * *p* < 0.05; ** *p* < 0.01. Significant differences are highlighted in bold.

**Table 3:** Scores and comparisons between the groups for the following dependent variables: impulsivity, activation, behavioral inhibition, and psychopathy.

Instrument	Variable	Group (*n*)	Mean (SD)	*F* (df; Sig.)
LSRP	Primary psychopathy	Control (23)	25.39 (5.255)	9.842 (3, 68; *p* = 0.000) *
		CRD (13)	34.77 (7.981)	
		CRD+SCZ (21)	31.95 (5.527)	
		CRD+APD (16)	34.25 (6.202)	
	Secondary psychopathy	Control (23)	17.26 (3.063)	15.474 (3, 68; *p* = 0.000) *
		CRD (13)	21.23 (7.585)	
		CRD+SCZ (21)	26.29 (5.100)	
		CRD+APD (16)	26.44 (4.953)	
PCL-R	Interpersonal/affective factor	Control (21)	28.14 (2.762)	103.019 (3, 63; *p* = 0.000) *
		CRD (11)	38.09 (7.217)	
		CRD+SCZ (19)	33.53 (3.533)	
		CRD+APD (16)	57.06 (7.750)	
	Social deviation factor	Control (21)	20.43 (1.076)	230.574 (3, 63; *p* = 0.000) *
		CRD (11)	31.82 (4.238)	
		CRD+SCZ (19)	36.74 (4.175)	
		CRD+APD (16)	58.81 (4.175)	
	Total score	Control (21)	20.52 (1.250)	230.495 (3, 63; *p* = 0.000) *
		CRD (11)	33.64 (4.178)	
		CRD+SCZ (19)	32.63 (4.284)	
		CRD+APD (16)	59.56 (5.573)	
SPSRQ	Sensitivity to punishment	Control (23)	8.26 (5.986)	6.369 (3, 68; *p* = 0.001) *
		CRD (13)	8.00 (4.583)	
		CRD+SCZ (21)	14.57 (5.363)	
		CRD+APD (16)	9.63 (5.084)	
	Sensitivity to reward	Control (23)	5.83 (3.950)	15.509 (3, 68; *p* = 0.000) *
		CRD (13)	10.92 (5.634	
		CRD+SCZ (21)	14.48 (4.214)	
		CRD+APD (16)	12.63 (4.193)	

Note: CRD, cocaine-related disorder; SCZ, schizophrenia; APD, antisocial personality disorder; SPSR, Sensitivity to Punishment and Sensitivity to Reward Questionnaire; LSRP, Levenson Self-Report Psychopathy Scale; PCL-R, Hare Psychopathy Checklist Revised. * *p* < 0.01.

## Data Availability

Data sharing is not applicable to this article.

## Supplementary Materials

The following are available online at https://www.mdpi.com/2076-3425/11/2/269/s1, Table S1: Characteristics of addictions, Table S2: Antipsychotics and other treatment drugs.
