# Supplementary material for: Prepulse Inhibition in Cocaine Addiction and Dual Pathologies
Source: Brain Sci. 2021 Feb 20;11(2):269. doi: 10.3390/brainsci11020269 (PMC7924364; doi:10.3390/brainsci11020269)
Supplement: Supplementary file 1 [file brainsci-11-00269-s001.pdf]

Table S1. Characteristics of addictions.

**Variables**	**CRD**	**CRD + SCZ**	**CRD + APD**	***F*/χ^2 (Sig.)**
**Cocaine**				
Addiction % (n)	100 (14)	100 (21)	100 (16)	-
Addiction severity	7.14 (1.83)	7.67 (2.72)	7.75 (1.77)	0.32 (0.724)
Age onset use	**23.71 (6.62)**	**18.81 (4.03)**	18.88 (6.13)	3.97 (0.025)*
Age onset addiction	**27.64 (7.92)**	**20.24 (4.13)**	**20.44 (7.33)**	6.64 (0.003)**
Weekly use	4-6 days	Less than a day	Daily	13.98 (0.058)
Route of use	**Intranasal**	**Intranasal**	**Smoked/Injected**	11.08 (0.022)*
**Nicotine**				
Addiction % (n)	92.3 (12)	100 (20)	93.3 (14)	1.50 (0.470)
Age onset use	14.85 (2.15)	14.90 (5.56)	12.93 (4.97)	0.89 (0.418)
Age onset addiction	15.75 (2.83)	16.67 (6.59)	13.33 (5.01)	1.70 (0.193)
Weekly use	Daily	Daily	Daily	4.95 (0.243)
Route of use	Smoked	Smoked	Smoked	2.75 (0.278)
**Alcohol**				
Addiction % (n)	50 (7)	66.7 (14)	56.3 (9)	1.02 (0.671)
Age onset use	15.40 (3.34)	13.57 (2.84)	12.64 (5.33)	1.43 (0.275)
Age onset addiction	**27.67 (9.13)**	20.54 (5.78)	**17.67 (5.72)**	4.26 (0.025)*
Weekly use	Daily	Daily	Daily	12.007 (0.298)
Route of use	Oral	Oral	Oral	11.22 (0.056)
**Cannabis**				
Addiction % (n)	57.1 (8)	76.2 (16)	75 (12)	1.68 (0.462)
Age onset use	**16.58 (2.10)**	15.29 (2.36)	**12.85 (4.54)**	4.62 (0.016)*
Age onset addiction	**19.80 (7.80)**	16.44 (2.39)	**14.08 (4.90)**	3.61 (0.037)*
Weekly use	**No use**	**2-3 days**/Daily	Daily	18.09 (0.031)*
Route of use	Smoked/**No use**	Smoked	Smoked	11.04 (0.007)**
**Amphetamines**				
Addiction % (n)	7.1 (1)	9.5 (2)	25 (4)	2.54 (0.346)
Age onset use	18.17 (1.83)	18.86 (5.17)	14.80 (3.11)	1.80 (0.199)
Age onset addiction	20.33 (4.50)	18.25 (1.70)	17 (2.82)	1.15 (0.359)
Weekly use	Less than a day	-	-	-
Route of use	Intranasal	-	-	-
**Heroine**				
Addiction % (n)	42.9 (6)	**19 (4)**	**87.5 (14)**	17.21 (<0.001)**
Age onset use	21.29 (5.49)	**25.80 (10.78)**	**14.79 (6.84)**	4.70 (0.019)*
Age onset addiction	22.14 (4.88)	26.50 (9.29)	17.54 (7.60)	2.65 (0.094)
Weekly use	No use/Less than a day	Less than a day	4-6 days	6.80 (0.313)
Route of use	Smoked	**Injected**	Smoked	17.08 (0.029)*
**Other opiates**				
Addiction % (n)	0	0	12.5 (2)	4.55 (0.166)
Age onset use	18 (-)	14 (-)	18.75 (13.93)	0.04 (0.955)
Age onset addiction	-	20 (-)	19 (13.92)	0.004 (0.953)
Weekly use	-	-	Daily	-
Route of use	-	-	Oral	-
**Sedatives**				
Addiction % (n)	14.3 (2)	**14.3 (3)**	**62.5 (10)**	12.29 (0.002)**
Age onset use	28.67 (10.01)	28.25 (6.23)	17.50 (6.88)	4.63 (0.029)*

Age onset addiction	29.67 (8.96)	26 (5.29)	17.50 (6.88)	4.25 (0.038)*
Weekly use	No use	2-3 days/Daily	Daily	10.20 (0.100)
Route of use	No use	Oral	Oral	7.03 (0.166)

Note: CRD: cocaine related disorder; SCZ: schizophrenia; APD: antisocial personality disorder. *p<0.05; **p<0.01. Significant differences are highlighted in bold. In sedatives age onset use and addiction Tukey post-hoc test does not reach significance.

Table S2. Antipsychotics and other treatment drugs.

Variables	CRD	CRD + SCZ	CRD + APD	F/χ^2 (Sig.)
**Antipsychotics**				
Treatment % (n)	25 (3)	**100 (19)**	**12.5 (2)**	50.48 (<0.001)**
Mean daily dose[1]	0.45 (0.39)	90.09 (52.09)	0.11 (0.08)	6.88 (0.006)**
**Quetiapine**				
Treatment % (n)	33.3 (4)	**73.7 (14)**	**16.7 (2)**	10.77 (0.004)**
Mean daily dose	381.25 (251.14)	514.28 (292.48)	100 (70.71)	2.09 (0.154)
**Olanzapine**				
Treatment % (n)	0	**26.3 (5)**	0	7.14 (0.047)*
Mean daily dose	-	24 (13.41)	-	-
**Paliperidone**				
Treatment % (n)	0	**78.9 (15)**	0	29.09 (<0.001)**
Mean daily dose	-	202.27 (96.47)	-	-
**Levomepromazine**				
Treatment % (n)	0	10.5 (2)	0	2.97 (0.334)
Mean daily dose	-	150 (70.71)	-	-
**Oxcarbazepine**				
Treatment % (n)	16.7 (2)	10.5 (2)	37.5 (6)	3.97 (0.190)
Mean daily dose	1200 (0)	1200 (0)	1200 (0)	-
**Methadone**				
Treatment % (n)	33.3 (4)	**0**	**56.3 (9)**	13.99 (0.001)**
Mean daily dose	72.50 (27.23)	-	81.11 (23.68)	0.33 (0.57)
**Suboxone**				
Treatment % (n)	8.3 (1)	10.5 (2)	25 (4)	1.98 (0.392)
Mean daily dose	8 (-)	12 (5.65)	8.5 (5.25)	0.32 (0.75)
**Benzodiazepines Treatment % (n)**	50 (6)	15.8 (3)	37.5 (6)	4.31 (0.119)
**Antidepressants Treatment % (n)**	33.3 (4)	10.5 (2)	12.5 (2)	3.06 (0.291)

Note: CRD: cocaine related disorder; SCZ: schizophrenia; APD: antisocial personality disorder. [1]Converted to chlorpromazine. *p<0.05; **p<0.01. Significant differences are highlighted in bold.
